# Microstructural and Residual Stress Homogenization of Titanium Sputtering Targets for OLED 6G Applications Through Controlled Rolling and Heat Treatment

**DOI:** 10.3390/ma18214965

**Published:** 2025-10-30

**Authors:** Leeseung Kang

**Affiliations:** Korea National Institute of Rare Metals, Korea Institute of Industrial Technology, Incheon 21655, Republic of Korea; leeseung@kitech.re.kr; Tel.: +82-32-226-1361

**Keywords:** Titanium, sputtering target, microstructural homogeneity, residual stress relaxation

## Abstract

The optimization of the microstructural homogeneity and residual stress distribution in Ti sputtering targets for OLED 6G applications is essential for improving dimensional stability, durability, and deposition performance. Herein, 3N Ti plates were hot-rolled at 730 °C and then annealed at 600 °C and 700 °C for different durations to investigate the effects of annealing parameters on microstructural evolution and stress relaxation. X-ray diffraction analysis revealed that hexagonal α-Ti with progressive development of the (002) orientation was produced during annealing under all the conditions. Electron backscatter diffraction analyses showed that short-time annealing at 600 °C (≤30 min) generated heterogeneous grains, high dislocation density, and mixed grain boundary character, whereas extended annealing (≥60 min) produced a more uniform microstructure. However, residual stress differences between the plate center and edge remained significant under this condition. Conversely, annealing at 700 °C promoted progressive recrystallization, as indicated by increased high-angle grain boundary fractions and decreased kernel average misorientation values, and facilitated grain growth stabilization across the plate. Prolonged annealing improved microstructural and residual stress uniformity significantly, and near-complete homogenization was achieved after 5 h. These findings demonstrate that annealing at 700 °C for sufficient time is optimal for producing homogeneous microstructures and uniform residual stress distributions, providing valuable guidelines for Ti sputtering target processing.

## 1. Introduction

The display industry has grown steadily alongside rapid technological advances. In particular, the performance of flat-panel displays (FPDs), such as thin-film-transistor liquid-crystal displays (TFT-LCDs) and organic light-emitting diode displays (OLED displays), has been significantly enhanced by breakthroughs in integrated circuit (IC) technology [1,2]. These advances have increased the demand for metallic materials used in thin-film fabrication and China has recently emerged as the dominant market for the thin-film industry, thereby accelerating related technologies [1].

To fabricate state-of-the-art display devices, various metal thin films are typically deposited using magnetron sputtering because of their versatility and industrial scalability [3,4,5]. Sputtering is a physical vapor deposition (PVD) method in which energetic ions bombard a target and eject atoms on a substrate to form thin films, which can critically affect the quality and properties of the deposited films [5,6,7]. Various metals, particularly Mo, Ti, TiN, Mo-Ti, and Cu, and their alloys have been widely used [8,9,10,11,12].

In particular, Ti is an essential material in TFT-LCDs and OLED displays, where it serves as a barrier layer for Al and Cu interconnects and as a capping layer that suppresses hillock formation and shields the lines from etching attack during IC fabrication [13,14,15,16]. Ti sputtering targets used in TFT-LCDs and OLED displays should satisfy the following conditions:

1. They should have high purity exceeding 3N (≥99.9%) to reduce particulate contaminations and prevent abnormal discharges during sputtering.
2. They must exhibit high density and a homogenous microstructure. Importantly, they should be free of non-uniform macro patterns to preserve film uniformity.
3. The average grain size should be under 70 μm, with grain-sized variations maintained below 20%, and crystallographic orientation spread limited to under 10%.
4. Adequate mechanical strength is required to ensure stable operation during high-rate sputtering without cracking or structural failure.

The crystallographic properties such as crystal structure, average grain size, crystallographic texture attributes both the sputtering process and the properties of the deposited thin films. For example, the hexagonal close-packed crystal structure of Ti induces a pronounced crystallographic texture, particularly strong basal or prismatic orientations [17], which significantly influences the angular distribution of ejected atoms during sputtering, thereby playing a pivotal role in film uniformity [18,19]. Additionally, a finer grain size accelerates the sputtering rate, enhances the uniformity of the film thickness, and suppresses particle contamination and abnormal discharge. To achieve these required properties, rolling and post heat treatment process should be necessitated [20].

Inaguma et al. applied multipass rolling deformation to various metals such as Mo, Al, Ta, Ti, and Ni, followed by heat treatment to produce refined and homogeneous microstructures. The crystallographic texture was carefully controlled to maintain {200} or {222} plane intensities within 15–80% of the total orientation distribution. Enhanced sputtering throughput and improved film thickness uniformity were successfully achieved using the strategic manipulation of grain refinement and preferred orientation [21].

Tsukamoto fabricated a 99.9995% (5N5) high-purity Ti sputtering target through successive hot forging, warm forging, cold rolling, and low-temperature annealing. This process achieved an average grain size below 10 µm, with the grain-size variation maintained under 20%, thereby successfully eliminating macro-shape defects and yielding a target that exhibited an outstanding thickness uniformity of 2.2% [22].

The effect of target microstructure on the deposition stability and uniformity of a 3N Ti sputtering target was elucidated by Yang et al. This study clarified the manner in which the target microstructure governs early erosion and discharge stability. As the etch depth and plasma density increased, sputtering progressed from defect-preferential erosion to grain-boundary–selective sputtering, then to closely packed grain-surface sputtering; the cross-sectional grain size increased (from 112 to 139 µm) and correlated strongly with sputtered-surface roughness (r = 0.802). This study emphasizes that the grain size and grain boundary (GB) character of the target affect the racetrack morphology and, by extension, the uniformity and stability of the sputtering process [23].

Although previous studies have extensively addressed grain size control and uniform grain size distribution through various rolling processes and heat-treatment parameters, the homogenization of residual stress, which is a critical factor governing the performance and service life of large-area sputtering targets, has received relatively less attention [20,24]. Although several studies have noted that non-uniform internal stresses induce microstructural heterogeneity and have attempted localized stress migration, thorough investigations into the systematic control and quantitative assessment of residual stress distribution across large-area targets remain notably scarce [25].

Accordingly, this study developed optimized rolling and heat treatment processes for large-area Ti sputtering targets used in display applications, with the dual objectives of stabilizing the microstructure and homogenizing the residual stress. To the best of our knowledge, this work represents the first comprehensive approach that simultaneously addresses uniform grain refinement and systematic control of the residual-stress distribution throughout large-area sputtering targets.

## 2. Materials and Methods

3N Ti plates with dimensions of 30 × 27 × 10 mm^3^ were used as the starting materials. The Ti plates were subjected to hot forging and annealing conditions. Before hot rolling, these plates were preheated at 600 °C for 1 h and consequently rolled to a thickness of 2.5 mm after 7 passes, corresponding to a 75% reduction rate. The roll diameter was Ф90 mm and Ф220 mm for both the working and backup rolls, respectively, and the roll length was 200 mm. Before performing crystallographic characterization, a 2 × 2 × 10 mm^3^ sample was fabricated and polished using a cross-section polisher (CP, JEOL, IB-19510, Japan) for 5–6 h in an Ar environment at 6.5 kV. The samples were observed using a scanning electron microscope (SEM, JEOL, JSM-7100F, Japan) equipped with an electron backscatter diffraction (EBSD) system, specifically a field-emission scanning electron microscope (FE-SEM). Subsequently, the EBSD data were analyzed using the HKL Technology Channel 5 program (Oxford Instruments, UK). The sin^2^ψ method involved the selection of the (101) lattice plane with 2θ of 40.2°, using the following parameters: a 2θ range of 36–44°, a tube voltage of 40 kV, a current of 35 mA, Cr–Kα radiation at 0.05° increments, and a scan time of 40 s. All measurements were performed in triplicate.

## 3. Results and Discussion

**Figure 1:**
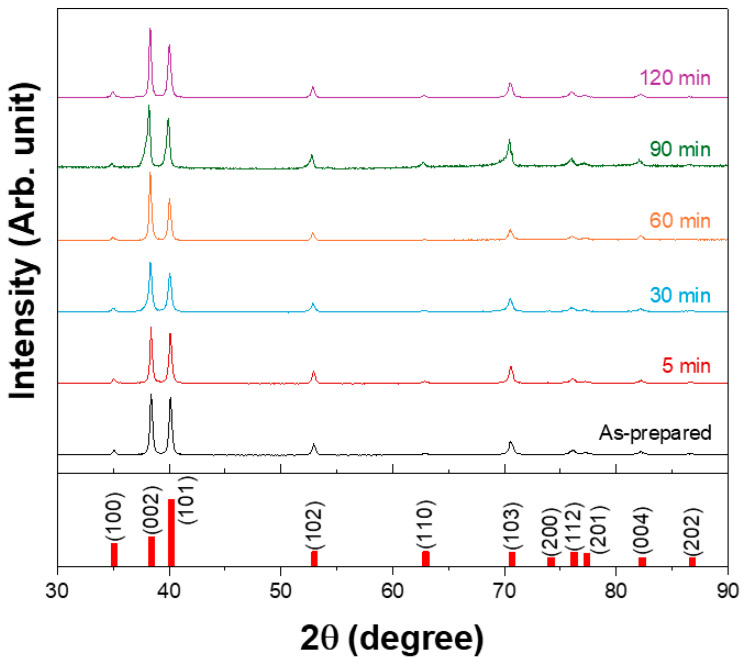
XRD patterns of the plate after hot rolling at 730 °C and subsequent heat treatment at 600 °C for different durations.

After hot rolling the 3N Ti plate at 730 °C, subsequent heat treatments were performed at 600 °C for various durations to obtain a homogeneous microstructure and stabilize the residual stress distribution. Figure 1 shows the X-ray diffraction (XRD) patterns of the rolled plates annealed at 600 °C for different durations. All the heat treatment conditions resulted in the formation of hexagonal structured α-Ti. However, with increasing annealing duration, the (002) and (004) planes became more pronounced, indicating the development of a basal-type texture by rolling and annealing. Similar strengthening of the basal texture of commercially pure Ti sheets using industrially feasible rolling and annealing processes has been reported recently [26,27].

Band contrast (BC) map analyses revealed that annealing for 5–30 min at 600 °C did not cause grain homogenization, whereas more uniform microstructures were obtained after ≥60 min (Figure S1). GB (Figure S2) and kernel average misorientation (KAM) maps (Figure S3) further showed that short-time annealing retained high dislocation densities and a mixture of low-angle grain boundaries (LAGBs) and high-angle grain boundaries (HAGBs), whereas prolonged annealing promoted the conversion from LAGBs to HAGBs and stabilized GBs, which is consistent with previous studies of static recrystallization (SRX) and texture evolution in Ti alloys [24,28]. These observations suggest that an insufficient annealing time fails to fully homogenize the grains.

During hot-rolling of 3N Ti, multiple passes increased the dislocation density, resulting in higher stored energy within the material. For sputtering target fabrication, high reduction ratios and multiple rolling passes are inevitable, and the strain imposed during each pass directly influences the grain size and uniformity. These microstructural variations, in turn, affect the target performance.

**Figure 2:**
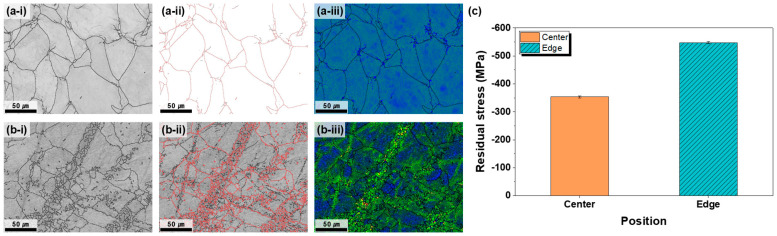
Comparison of the microstructures (**a-i**–**a-iii**) at the center and (**b-i**–**b-iii**) at the edge of the plate after hot rolling at 730 °C and subsequent heat treatment at 600 °C for 1 h, showing (**i**) band contrast, (**ii**) grain boundary (GB) map—red lines indicate low-angle grain boundaries (LAGBs) and black lines indicate high-angle grain boundaries (HAGBs)—and (**iii**) kernel average misorientation (KAM) map, where blue regions represent areas of low KAM and light-green regions correspond to areas of high KAM. (**c**) Residual-stress comparison between the center and edge regions.

According to the BC map analysis (Figure S1), the grain size of the plates annealed at 600 °C for 1 h or longer remained below 70 μm, thereby meeting the requirements of commercial OLED 6G sputtering targets. However, to further evaluate the spatial uniformity, the microstructures at the plate center and edge were compared (Figure 2). The BC maps indicate that the center contained relatively uniform equiaxed grains, whereas the edges exhibited mixed fine and coarse grains and partially recrystallized regions. GB maps confirmed a predominance of HAGBs at the center and higher LAGB fractions at the edge, reflecting inferior microstructural homogeneity. The KAM maps further demonstrate that the center region had low dislocation accumulation and stable energy states, whereas the edge retained higher strain and accumulated dislocations, reflecting a higher energy state. These results indicate that the strain heterogeneity introduced during rolling causes uneven SRX, particularly at the plate edge, resulting in overall microstructural non-uniformity after annealing [24,27]. Post-rolling heat treatment relaxes or eliminates the residual stresses by enabling defect rearrangement and microstructural stabilization. During heat treatment, heating to an adequate temperature and maintaining it for a sufficient duration enables defect rearrangement and microstructural stabilization, thereby reducing the stress. Annealing parameters such as temperature and time directly influence the stress relaxation process; insufficient conditions fail to relieve defects, whereas excessive temperatures may cause oversoftening or abnormal grain growth. For sputtering targets, a uniform residual stress distribution is essential to improve the dimensional stability, durability, and target lifetime and to ensure deposition quality. Residual stress comparison between the center and edge of the plate annealed at 600 °C for 1 h revealed a large difference of 198.2 MPa (Figure 2c). The higher compressive residual stress at the edge is attributed to strain localization during rolling, differences in the recrystallization behavior, and variations in the cooling rate, as shown in Figure 2(a-i,a-ii,a-iii,b-i,b-ii,b-iii). Even when the annealing time at 600 °C was extended to 1.5 h and 2 h, the difference in the residual stress between the center and edge of the plate did not decrease appreciably (Figure S4), indicating that longer durations at this temperature were insufficient for stress homogenization. Mechanistically, the persistent center and edge residual-stress difference after annealing at 600 °C can be rationalized by the competition between recovery and static recrystallization (SRX) in α-Ti. At 600 °C (≈0.45 T_m_), recovery processes—dislocation rearrangement and sub grain coarsening—predominate, which lowers the overall dislocation density but does not fully remove the heterogeneity in stored energy inherited from rolling. Consistent with this, the edge region exhibits higher KAM values and a larger fraction of LAGBs than the center, indicating incomplete boundary stabilization and limited nucleation of new, strain-free grains. The resulting heterogeneous microstructure sustains internal elastic incompatibility and thus preserves a finite residual-stress gradient across the plate [24,25,28]. This behavior is consistent with the temperature-dependent relaxation regimes reported for commercially pure Ti, in which effective stress relaxation generally requires temperatures higher than the recrystallization threshold [25,29]. Therefore, to provide additional driving energy for recrystallization and stress relaxation, the annealing temperature was increased to 700 °C, and the effects of time on microstructural and residual-stress uniformity were examined.

**Figure 3:**
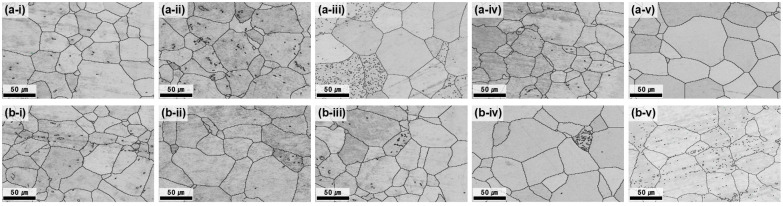
BC maps of the plate after hot rolling at 730 °C and subsequent heat treatment at 700 °C. (**a-i**–**a-v**) depict the center region and (**b-i**–**b-v**) the edge region, each corresponding to heat treatment times of 1, 2, 3, 4, and 5 h.

**Figure 4:**
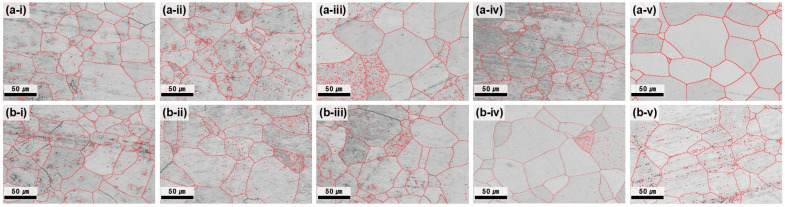
GB maps of the plate after hot rolling at 730 °C and subsequent heat treatment at 700 °C. (**a-i**–**a-v**) depict the center region and (**b-i**–**b-v**) the edge region, each corresponding to heat treatment times of 1, 2, 3, 4, and 5 h.

At 700 °C, all the conditions satisfied the grain-size requirement (<70 µm); however, the center–edge grain-size difference was still notable at ≤3 h (Figure 3). As the annealing time increased, the average grain size difference progressively decreased, reflecting the enhanced homogenization of grain growth and the reduction in strain localization effects. After 5 h of annealing, the difference between the center and edge was reduced to 1.12 μm, demonstrating nearly complete microstructural uniformity. The GB map analysis (Figure 4) confirmed that the HAGB fractions increased with annealing time, indicating progressive recrystallization. In particular, after 5 h, the GBs were predominantly HAGBs, indicating complete recrystallization. These results highlight that prolonged annealing at 700 °C effectively reduced dislocation density and stabilized GB structures, thereby maximizing microstructural uniformity. The time-dependent transition from LAGBs to HAGBs in this study is similar to previous observations in β-Ti alloys, where longer annealing times drive gradual SRX and yield a more uniform microstructure and a more stable texture [28].

**Figure 5:**
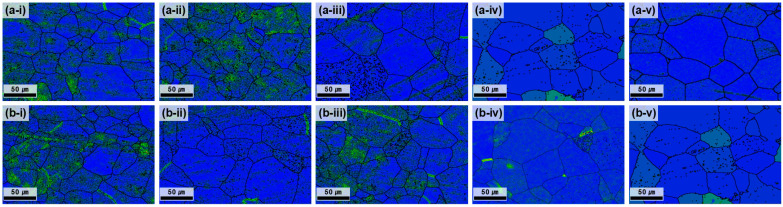
KAM maps of the plate after hot rolling at 730 °C and subsequent heat treatment at 700 °C. (**a-i**–**a-v**) depict the center region and (**b-i**–**b-v**) the edge region, each corresponding to heat treatment times of 1, 2, 3, 4, and 5 h.

KAM maps of samples annealed at 700 °C for various durations (Figure 5) revealed that 1–3 h annealing retained relatively high KAM values across different regions, indicating the presence of dislocations and incomplete strain release. After 4 h, the KAM values decreased significantly, reflecting reduced dislocation density and internal strain. At 5 h, the effect was maximized with an overall stabilization of the energy states.

**Figure 6:**
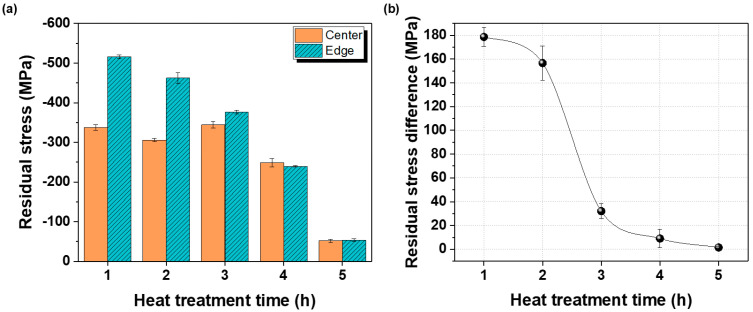
(**a**) Comparison of residual stresses at the center and edge of the plate after hot rolling at 730 °C and subsequent heat treatment at 700 °C for different durations, and (**b**) variation in residual stress within the plate under the same conditions.

Residual stress results for the center and edge regions after annealing at 700 °C are shown in Figure 6. At annealing times ≤ 3 h, the edge retained higher compressive residual stress than the center owing to higher strain accumulation during rolling. However, with increasing annealing time, the residual stress difference between the center and edge decreased, resulting in more uniform stress distributions. After 4 h, the residual-stress differences were nearly eliminated, and after 5 h, both residual-stress minimization and complete uniformity across the plate were achieved.

## 4. Conclusions

In this study, the microstructural evolution and residual stress behavior of 3N Ti plates subjected to hot rolling at 730 °C followed by heat treatment under various conditions were systematically investigated. The key findings are summarized as follows:

1. Phase and texture development

XRD analysis confirmed that hexagonal α-Ti was formed under all heat treatment conditions. With increasing annealing temperature, a pronounced development of the (002) plane was observed, indicating a strong rolling- and annealing-induced texture. This texture evolution has potential implications for the sputtering performance, particularly in relation to the preferential growth of the (002) plane.

2. Effect of heat treatment temperature of 600 °C

EBSD analyses demonstrated that short-time annealing at 600 °C (≤30 min) was insufficient to homogenize the microstructure, as grains remained heterogeneous with high dislocation densities and mixed LAGBs/HAGBs. With prolonged annealing (≥60 min), grain refinement and boundary stabilization were achieved, resulting in a more homogeneous microstructure that satisfied the grain size requirement (<70 μm) for OLED sputtering targets. However, residual stress measurements revealed persistent disparities between the center and edge regions, even after extended annealing at 600 °C. Therefore, although microstructural uniformity can be obtained under these conditions, residual stress uniformity cannot be ensured.

3. Effect of heat treatment temperature of 700 °C

Raising the annealing temperature to 700 °C promoted recrystallization and grain growth stabilization across the plate. With increasing annealing time, the microstructural uniformity progressively improved. After 5 h, the center and edge regions exhibited nearly identical grain sizes and GB character distributions. Concurrently, the residual stress differences between the center and edge decreased with annealing time and became negligible after 4–5 h. Therefore, both microstructural uniformity and residual stress uniformity were achieved under these conditions.

In summary, hot rolling at 730 °C followed by annealing at 700 °C for 5 h was identified as the most effective condition for producing a 3N Ti sputtering target with homogeneous microstructures and uniform residual stress distributions. These results provide valuable guidelines for optimizing Ti sputtering target processing to enhance dimensional stability, durability, and sputtering performance in display applications.

## Data Availability

The original contributions presented in this study are included in the article/Supplementary Material. Further inquiries can be directed to the corresponding author.

## Supplementary Materials

The following supporting information can be downloaded at: https://www.mdpi.com/article/10.3390/ma18214965/s1, Figure S1: BC maps of the plate after hot rolling at 730 °C and subsequent heat treatment at 600 °C for (a) 5 min, (b) 30 min, (c) 60 min, (d) 90 min, and (e) 120 min; Figure S2: GB maps of the plate after hot rolling at 730 °C and subsequent heat treatment at 600 °C for (a) 5 min, (b) 30 min, (c) 60 min, (d) 90 min, and (e) 120 min; Figure S3: KAM maps of the plate after hot rolling at 730 °C and subsequent heat treatment at 600 °C for (a) 5 min, (b) 30 min, (c) 60 min, (d) 90 min, and (e) 120 min; Figure S4: Comparison of residual stresses measured at the center and edge of the plate after hot rolling at 730 °C and subsequent heat treatment at 600 °C for different times.
