# Supplementary material for: Microstructural and Residual Stress Homogenization of Titanium Sputtering Targets for OLED 6G Applications Through Controlled Rolling and Heat Treatment
Source: Materials (Basel). 2025 Oct 30;18(21):4965. doi: 10.3390/ma18214965 (PMC12608695; doi:10.3390/ma18214965)
Supplement: Supplementary file 1 [file materials-18-04965-s001.zip › materials-3958853-supplementary.pdf]

Supporting Information

Microstructural and Residual Stress Homogenization of Titanium Sputtering Targets for OLED 6G Applications Through Controlled Rolling and Heat Treatment

Leeseung Kang

Korea National Institute of Rare Metals, Korea Institute of Industrial Technology, Incheon 21655, Republic of Korea; leeseung@kitech.re.kr

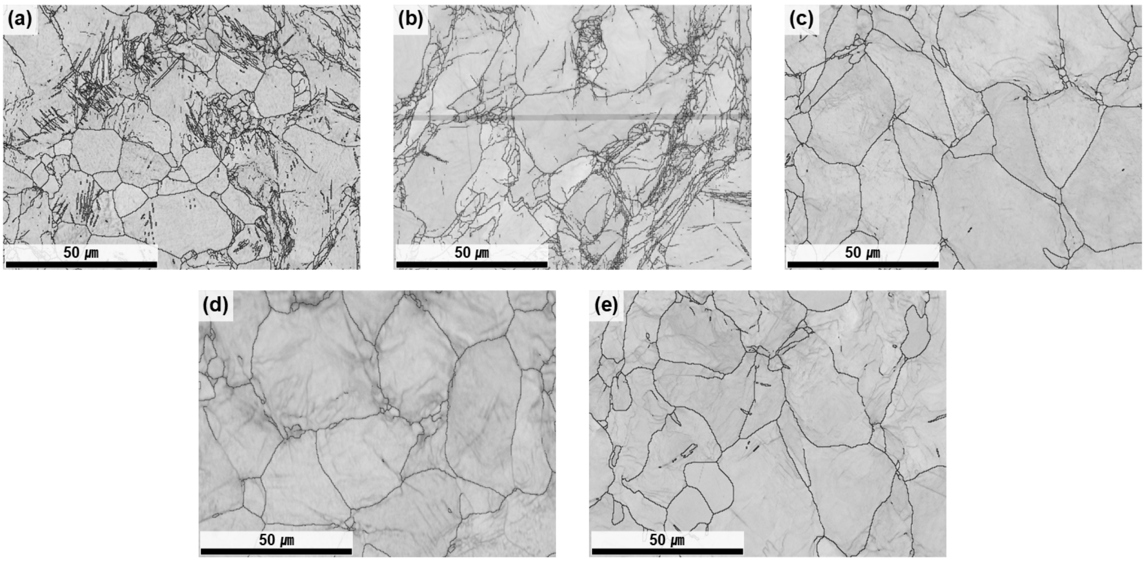

Figure S1. BC maps of the plate after hot rolling at 730 °C and subsequent heat treatment at 600 °C for (a) 5 min, (b) 30 min, (c) 60 min, (d) 90 min, and (e) 120 min.

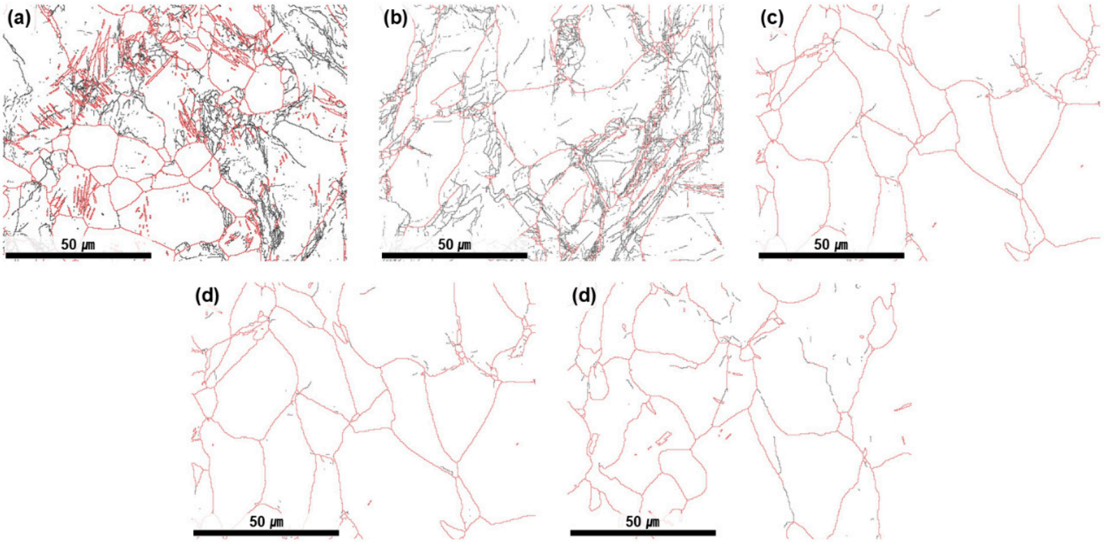

Figure S2. GB maps of the plate after hot rolling at 730 °C and subsequent heat treatment at 600 °C for (a) 5 min, (b) 30 min, (c) 60 min, (d) 90 min, and (e) 120 min.

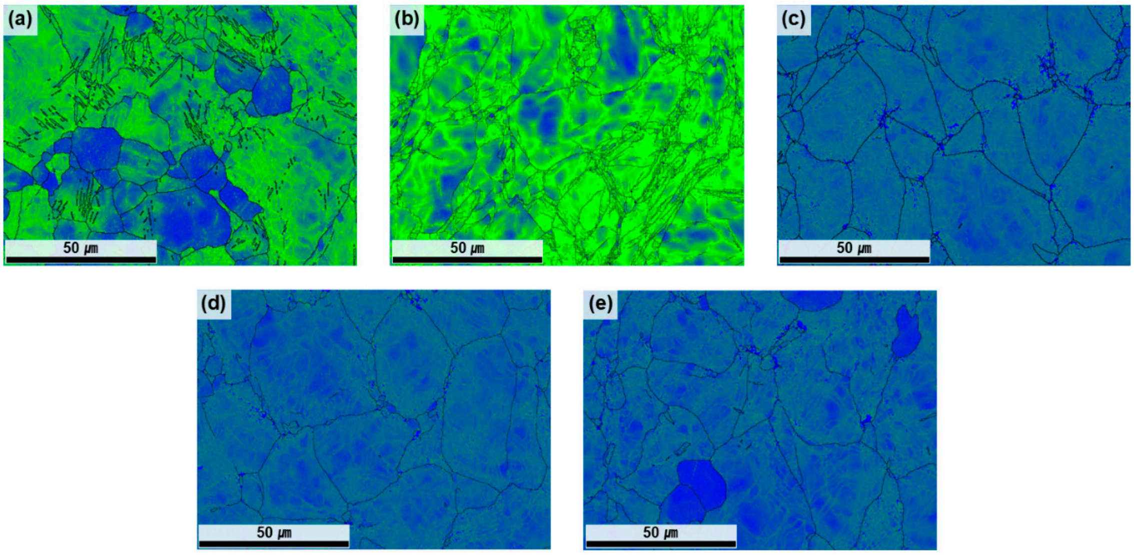

Figure S3. KAM maps of the plate after hot rolling at 730 °C and subsequent heat treatment at 600 °C for (a) 5 min, (b) 30 min, (c) 60 min, (d) 90 min, and (e) 120 min.

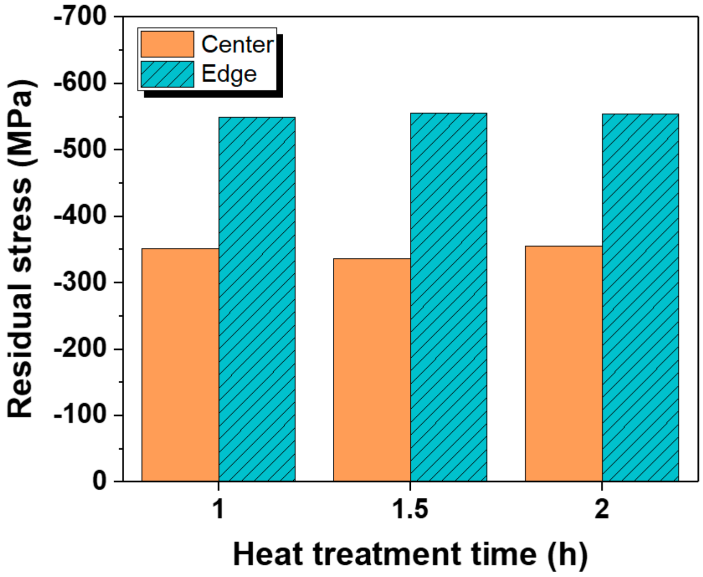

Figure S4. Comparison of residual stresses measured at the center and edge of the plate after hot rolling at 730 ℃ and subsequent heat treatment at 600 ℃ for different times.
